# Response by Hadid et al to “Hemostatic Nets in Facelifts: A Systematic Review and Meta-Analysis of Postoperative Complications and Patient Outcomes” by Caimi et al

**DOI:** 10.1093/asjof/ojaf146

**Published:** 2026-02-16

**Authors:** Karam Hadid, Forrest Bohler, Kongkrit Chaiyasate

We read with interest the recent article by Caimi et al, “Hemostatic Nets in Facelifts: A Systematic Review and Meta-Analysis of Postoperative Complications and Patient Outcomes.”^1^ While the topic is of clinical relevance, we identified several methodological concerns that we believe undermine the reliability of the authors’ conclusions.

Most notably, 2 of the included studies by Auersvald et al (2012 and 2014) appear to draw from overlapping patient populations.^2,3^ Both originate from the same surgeon and institution, cover overlapping study periods, and, most strikingly, report control groups of identical size (*n* = 120), identical mean age to 2 decimal places (55.40 years), and identical age range (33-77 years) as seen in the paper's Table 1.^1^ The probability of these parameters being identical in 2 independent control cohorts is extremely low, strongly suggesting that the same patients were included in both publications. Inclusion of these likely duplicate patients appears to have had a significant influence on the reported meta-analytic results. For example, 57.4% (651/1135) of patients contributing to the pooled hematoma rate in Figure 2, and as many as 89.1% (651/731) of those in the transient postoperative changes analysis in Figure 4, are likely duplicates from overlapping patient cohorts. If confirmed that these cohorts are overlapping, we respectfully suggest that the authors reanalyze their data in a response letter with one of the studies removed to provide an accurate and unbiased synthesis.

Further, the risk-of-bias assessment raises concerns. The authors applied the Newcastle-Ottawa Scale uniformly to all included studies, despite substantial variation in study design, including a randomized controlled trial, prospective-retrospective cohort studies, and an uncontrolled case series. The Newcastle-Ottawa Scale is not validated for randomized trials or case series, and its use in these contexts produces misleading quality scores and creates false equivalence between fundamentally different levels of evidence.^4^ Established, design-specific tools such as the Cochrane RoB 2 for randomized trials and JBI checklists for case series would have provided a more accurate and defensible evaluation of bias.^5^

Given these issues, particularly the apparent duplication of patient data, we urge caution in interpreting the reported findings. Addressing these concerns in future meta-analyses will be critical to ensuring that conclusions about the efficacy and safety of hemostatic nets are both robust and reproducible.

## References

[1] Caimi E, Pellicanò F, El Choueiri J, et al Hemostatic nets in facelifts: a systematic review and meta-analysis of postoperative complications and patient outcomes. Aesthetic Surg J Open Forum. 2025;7:ojaf082. doi: 10.1093/asjof/ojaf082 PMC12715502 41426293

[2] Auersvald A, Auersvald L, Biondo-Simões M. Hemostatic net: an alternative for the prevention of hematoma in rhytidoplasty. Rev Bras Cir Plástica. 2012;27:22–30. doi: 10.1590/S1983-51752012000100006

[3] Auersvald A, Auersvald LA. Hemostatic net in rhytidoplasty: an efficient and safe method for preventing hematoma in 405 consecutive patients. Aesthetic Plast Surg. 2014;38:1–9. doi: 10.1007/s00266-013-0202-5 23949130

[4] Gierisch JM, Beadles C, Shapiro A, et al Newcastle-Ottawa scale coding manual for cohort studies. Health Disparities in Quality Indicators of Healthcare Among Adults with Mental Illness [Internet]. Department of Veterans Affairs (US); 2014. 26065051

[5] Munn Z, Barker TH, Moola S, et al Methodological quality of case series studies: an introduction to the JBI critical appraisal tool. JBI Evid Synth. 2020;18:2127–2133. doi: 10.11124/JBISRIR-D-19-00099 33038125

